# Ionized and total serum magnesium in hemodialysis: predictors and variability. A longitudinal cross-sectional study

**DOI:** 10.1007/s10157-017-1494-6

**Published:** 2017-12-07

**Authors:** Rosaria Del Giorno, Hilary Riva, Gaetano Donato, Luca Gabutti

**Affiliations:** 1 Department of Internal Medicine and Nephrology, San Giovanni Regional Hospital, Bellinzona, Switzerland; 2 Department of Internal Medicine and Nephrology, Beata Vergine Regional Hospital, Mendrisio, Switzerland; 3 Division of Nephrology, Ospedale la Carità, Locarno, Switzerland; 4 Department of Internal Medicine and Nephrology, Bellinzona Regional Hospital, 6500 Bellinzona, Switzerland

**Keywords:** Ionized magnesium, Variability, Prediction formula, Inter-individual variability, Intra-individual variability, Seasonal variability

## Abstract

**Background:**

Ionized Magnesium (ion-Mg) represents the active biological fraction of the serum magnesium content. The assessment of total serum Mg (tot-Mg) might not accurately identify patients with hypo-or hyper-magnesaemie. In hemodialysis, serum tot-Mg levels in the upper part of the distribution, have been associated with reduced mortality and fewer vascular calcifications; thus, resulting in the tendency to increase the Mg concentration in the dialysate, traditionally set at 0.5 mmol/L.

**Methods:**

Single-center study in chronic hemodialysis patients, designed in two phases, cross-sectional and longitudinal, aimed to investigate: (1) the sensitivity for pathological values of ion-Mg compared to tot-Mg (2) the predictors of ion-Mg developing ad hoc equations; (3) the inter- and intra-individual variabilities of ion-Mg; and (4) the risk factors for hypermagnesemia. Tot-Mg, ion-Mg, and covariates of 42 hemodialysis sessions, in 42 patients during the cross-sectional phase and of 270 sessions in 27 patients in the longitudinal one were analysed.

**Results:**

Ion-Mg significantly correlates with tot-Mg: *β* = 0.52; *r* = 0.88, *p* < 0.001. Multiple linear regressions in normo- and hypo-albuminemic patients gave the following results: ion-Mg = tot-Mg/2-K^+^/50 + Ca^2+^/5-HCO^3−^/100 and ion-Mg = tot-Mg/2 + albumin/100. Ion-Mg showed a high temporal variability in the longitudinal phase (between months *p* < 0.001; winter vs. summer, *p* < 0.027). A high intra-individual variability was also found: coefficient of variation 0.116. Comparing patients with high and low intra-individual variability, we found: age 67 vs. 77 years; *p* < 0.001; urea 26.3 ± 0.5 vs. 21.2 ± 0.4 mmol/L, *p* < 0.001; nPCR 0.92 ± 0.1 vs. 0.77 ± 0.1 g/kg day, *p* < 0.001; PTH 46.3 ± 4 vs. 28.5 ± 3 pmol/L, *p* < 0.001.

**Conclusions:**

Ion-Mg can be useful in unmasking unrecognized hyper- and hypo-magnesemic and false hyper-magnesemic patients. Ion-Mg is characterized by high intra- and inter-individual variabilities particularly in younger women and those with better nutrition. Patients with greater variability could potentially be at risk if exposed to higher concentrations of magnesium in the dialysate. An interventional study, with controlled increase of magnesium concentrations in the dialysate has been planned.

## Introduction

Ionized Magnesium (ion-Mg) represents the active and the most abundant biological fraction of the serum magnesium [1, 2]. Nevertheless, despite having several limitations, the assessment of the total serum Mg (tot-Mg) concentration is the only test available in the clinical setting for the evaluation of magnesium level. Tot-Mg could, in particular, not accurately detect patients with pathological magnesium levels and it does not adequately reflect the whole Mg body content [3, 4]. In hemodialysis (HD) patients, some additional limitations in the reliability of tot-Mg measurement could contribute to a higher degree of variability and finally lead to misinterpretation of the magnesemic status (e.g. the renal function impairment influences magnesium homeostasis in particular via acid–base status and calcium–vitamin D axes alterations) [5].

The role of Mg in patients with chronic kidney disease (CKD) is an object of debate. It is well known that CKD patients with or without HD show a higher incidence of death and CV diseases [6]. This, in addition to the traditional and CKD-associated CV risk factors, can be partially attributable, to the calcifications of the large and middle arteries, typically seen in HD patients, and mainly related to mineral and bone metabolism disorders [7]. Experimental studies in vitro and in the animal models have shown that high extracellular Mg levels can have an inhibitory effect on the calcification processes involving both, mechanisms related to local and systemic mineralization inhibition and osteogenic differentiation [8–10]. Moreover, findings of large epidemiological studies have highlighted that serum magnesium values in the upper part of the normal distribution in HD patients are associated with higher survival rates: HR, 0.485; 95% CI 0.241–0.975; per 0.41 mmol/L (1 mg/dL) increase in total serum magnesium [11]. A J-shaped association between serum Mg levels and the odds ratio for all-cause mortality was also found and confirmed after appropriate adjustments for relevant clinical factors [12]. Furthermore, in the large cohort of the study “Atherosclerosis Risk in Communities” (ARIC), an independent association between low serum magnesium levels and incidental heart failure was reported [13]. Finally, studies aimed at analyzing the hemodynamic pattern, suggest that a higher dialysate magnesium concentration (0.75 instead of 0.50 mmol/L) could be a safe option to counteract intra-dialytic hypotension [14].

The possibility of obtaining both cardiovascular and hemodynamic benefits in HD patients by changing the dialysate Mg concentration is intriguing. To date HD guidelines do not recommend adapting the dialysate Mg concentration, routinely set at 0.50 mmol/L, even if concentrations between 0.25 and 1.00 mmol/L, have been tested in clinical studies [14, 15] showing that increasing dialysate Mg concentration could be beneficial.

The well-known significant intra-individual variability in serum tot-Mg could, however, affect the safety of increasing the Mg dialysate concentration in HD patients in an unrestricted way [14]. Even if the inter- and intra-individual variabilities in this setting has been investigated only in a few studies, ion-Mg could offer a reliable parameter [16].

It should be stressed that, despite the potential beneficial effects of magnesium levels in the upper normal range, hypermagnesemia could cause several side effects mainly related to electrocardiographic abnormalities, nerve conduction disturbances, pruritus, and bone metabolism and parathyroid gland function alterations, potentially contributing to both renal osteodystrophy and adynamic bone disease [17].

In the present study, designed in two phases (preliminary cross-sectional phase and longitudinal phase with 12 months of follow-up) we aimed: (1) to compare the ion-Mg with the serum tot-Mg investigating whether tot-Mg may over- or under-estimate the incidence of hypo- and hyper-magnesemia; (2) to explore predictors of tot-Mg and ion-Mg and to derive and subsequently validate a simple formula to estimate ion-Mg (ion-Mg is neither easily nor routinely measured in most laboratories); (3) to investigate the variability of pre- and post-HD ion-Mg through a cross-sectional and longitudinal time-series, (4) to explore risk factors for punctual hyper-magnesemic values.

## Methods

### Study design, setting, patients

This study was conducted between June 2014 and July 2015 at the Dialysis Unit of the “La Carità Hospital” of Locarno (Locarno, Switzerland). The study was designed as a single-center, two-phase cross-sectional one; with 1 month observational (in June) and 12 month longitudinal phases and was performed in adult patients with End Stage Renal Disease treated with conventional HD. The study protocol was approved by the local ethics committee (Swiss Ethics Committee no. 2794).

All the participants gave written informed consent.

**Fig. 1:**
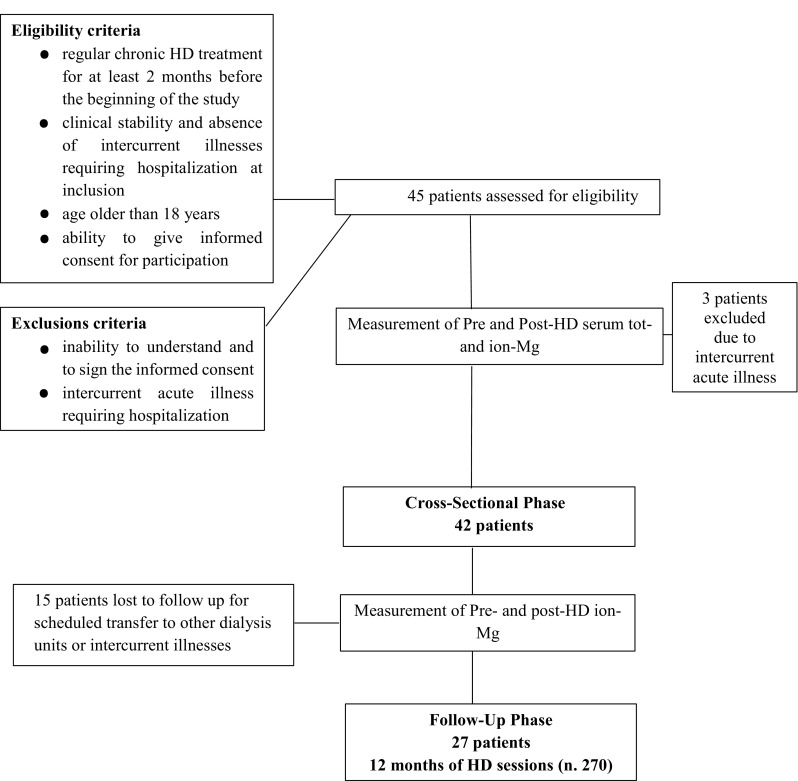
Flow chart of the study

Eligibility criteria for enrollment included: (1) regular chronic HD treatment for at least 2 months before the beginning of the study, (2) clinical stability and absence of intercurrent illnesses during the study; (3) age older than 18 years; (4) ability to give informed consent for participation. The exclusions criteria were: (1) inability to understand and to sign the informed consent; (2) intercurrent acute illness requiring hospitalization during the study period. Data of 42 HD patients aged ≥ 18 years on a regular thrice weekly HD-program were recorded in the cross-sectional 1 month phase and of 27 HD patients in the follow-up phase (see Fig. 1 flow diagram of the study).

All dialysis sessions were performed with standard Mg dialysate concentration of 0.5 mmol/L. The dialysate solutions were otherwise identical for the calcium concentration set at 1.25 mmol/L.

Overall, we analyzed the results of 1 month of HD-sessions (*N*: 42) for the cross-sectional phase and 12 months of HD sessions (*N*: 270) for the longitudinal one.

### Measurements and data collection

Demographic, renal and basic laboratory parameters were recorded in both phases and included age, gender, comorbidity, medications, ultrafiltration, pre‐ and post‐HD body weights and HD-session duration. Pre- and post‐HD serum urea levels were used for the calculation of the normalized protein catabolic rate (nPCR). In the cross-sectional phase the following parameters on pre‐ and post‐HD blood samples were also determined: serum tot-Mg, ion-Mg, pH, serum bicarbonate (HCO^3−^), ionized calcium (Ca^2+^). Serum potassium (K^+^), sodium (Na^+^), phosphate, parathormone (PTH), 25-OH vitamin D and hemoglobin (Hb) were measured on pre-HD samples only. In the follow-up phase the monitoring was limited to: ion-Mg (pre- and post-HD); PTH; Ferritin and 25 OH-vitamin D.

For the measurement of ion-Mg we used an ionometer (Microlyte 6 Analyzer, Kone Instruments, Espoo, Finland). All hematological parameters were determined using standard techniques in the central laboratory of the Ente Ospedaliero Cantonale, Switzerland.

### Statistical analysis

Descriptive statistics are presented as median (interquartile range, IQR), or as mean ± standard deviation (SD) for continuous, and as numbers and percentages for categorical variables. Continuous and categorical variables were compared using *t* tests or Wilcoxon rank sum tests and *χ* ^2^ tests, respectively. Subjects were classified as hyper-, hypo-, or normo-magnesemic according to the reference range values of serum tot-Mg and ion-Mg (0.65–1.05; and 0.45–0.67 mmol/L respectively). In the cross-sectional phase stepwise, multiple linear regressions with backward elimination were performed to determine the effect of the potential explanatory variables, considering ion-Mg as the dependent one. Based on the original model, total covariates significantly associated with the dependent variable at *p* < 0.05, were considered. The numeric contribution of variables of interest was then assessed and the final regression equations, algebraically simplified, were constructed.

In the longitudinal phase of the study, the outcome of interest was the between- and intra-patient long-term variabilities of ion-Mg. The longitudinal trend of ion-Mg was graphically depicted and the within-subject variability was evaluated using a coefficient of variation (standard deviation and mean).

Data analysis was performed using R statistical software (http://www.r-project.org) and STATA 14 (Stata Corporation, College Station, TX). Statistical significance for all outcomes was set at *p* ≤ 0.05.

## Results

Data of 42 and of 26 patients were analyzed, in the cross-sectional and longitudinal-follow-up phase, respectively. Demographic data, clinical characteristics and laboratory results are shown in Table 1. In both study phases, gender, age, renal parameters, nutritional status and ion-Mg values were similar (see Table 1 for details).

**Table 1:** Characteristics of the study population

Cross-sectional phase (patients no. 42)	
Age (years)	72 (63–81)
Males (%)	43%
Time HD (h)	4 (3.5–4)
Ultrafiltartion (mL/h)	2300 (1300–2800)
Urea pre HD (mmol/L)	21.3 (16.7–28.3)
Urea post HD (mmol/L)	5.3 (3.8–6.9)
nPCR (g/kg)	0.79 (0.64–0.95)
Total Mg pre-HD (mmol/L)	0.93 (0.83–1.02)
Total Mg post-HD (mmol/L)	0.83 (0.76–0.87)
Ionized Mg pre-HD (mmol/L)	0.58 (0.52–0.63)
Ionized Mg post-HD (mmol/L)	0.55 (0.51–0.58)
Phosphates (mmol/L)	1.55 (1.35–1.98)
Albumin (g/L)	34 (32–38)
PH pre HD	7.35 (7.32–7.38)
PH post HD)	7.46 (7.41–7.51)
HCO^3−^ pre HD (mmol/L)	22 (20–24)
HCO^3−^post HD (mmol/L)	28 (26–29)
Ionized Ca^++^ pre HD (mmol/L)	1.18 (1.1–1.21)
Ionized Ca^++^ post HD (mmol/L)	1.16 (1.1–1.21)
K^+^ pre HD (mmol/L)	4.7 (4.2–5.4)
Na^+^ pre HD (mmol/L)	139 (136–141)
Hb (g/L)	116 (105–127)
PTH (pmol/L)	30 (18.5–47.6)
25-OH-vitamin-D (ng/mL)	41.9 (28.2–52.6)
Longitudinal phase (patients no. 27)	
Age (years)	71 (62–81)
Males (%)	48.1%
Urea pre HD (mmol/L)	23 (20–28.2)
Urea post HD (mmol/L)	5.5 (4.3–6.8)
nPCR (g/kg)	0.84 (0.73–0.97)
Ion-Mg pre-HD (mmol/L)	0.59 (0.55–0.63)
Ion-Mg-Post HD (mmol/L)	0.53 (0.50–0.56)
Weight (kg)	70.8 (60.2–80.4 )
PTH (pmol/L)	33.7 (18.5–48.2)
Ferritin (ng/L)	479 (280–755)
25-OH-vitamin-D (ng/mL)	44.2 (34.5–49.7)

Continuous variables are expressed as median (IQR)

**Fig. 2:**
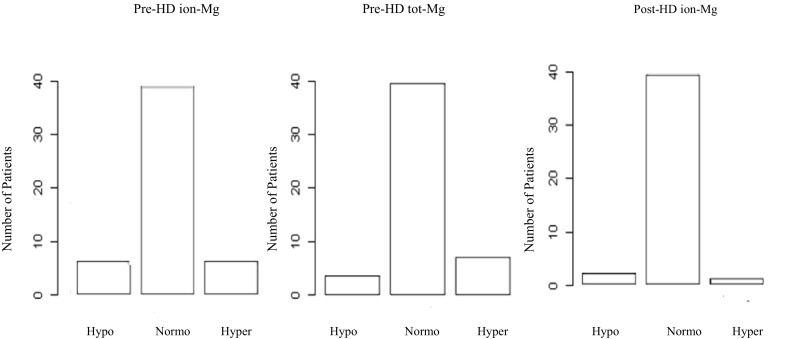
Hypo-, normo- and hyper-magnesemic patients in the cross-sectional phase. Amount of patients, ipo-, normo- and hyper-magnesemic according to reference value ranges for ion-Mg and serum tot-Mg. A significant difference between the rate of patients considered hypo-magnesemic was seen (hypo serum tot-Mg vs. hypo ion-Mg; *p* value < 0.05)

We investigated the rate of normo-, hypo- and hyper-magnesemic patients, based on reference range values of serum tot-Mg and ion-Mg. In the cross-sectional phase, considering the values of pre-HD ion-Mg, patients were normo-, hypo- and hyper-magnesemic in 76, 12 and 12% of the cases respectively. Based on the value of serum tot-Mg the percentages were instead: 80 for normo-, 6 for hypo- and 14 for hyper-magnesemic. Discrepancies between the patients detected and a significant difference between the rate of hypo-magnesemic patients using the two methods was found (*p* < 0.05) (Fig. 2). Considering the well-known association between proton pump inhibitors (PPI) use and serum magnesium levels [18], and the potential risks related to hypomagnesemia in hemodialysis patients, the association between magnesium values and the use of PPI was investigated post hoc. No differences across groups (PPI users vs. non-users) were found: ion-Mg pre-HD 0.57 mmol/L (0.54–0.61) vs. 0.58 mmol/L (0.53–0.62), *p* value 0.545; tot-Mg pre-HD: 0.94 mmol/L (0.87–1.00) vs. 0.93 (0.85–1.00) *p* value 0.429; ion-Mg- post-HD 0.55 mmol/L (0.53–0.56) vs. 0.55 (0.52–0.57) *p* value 0.484.

**Fig. 3:**
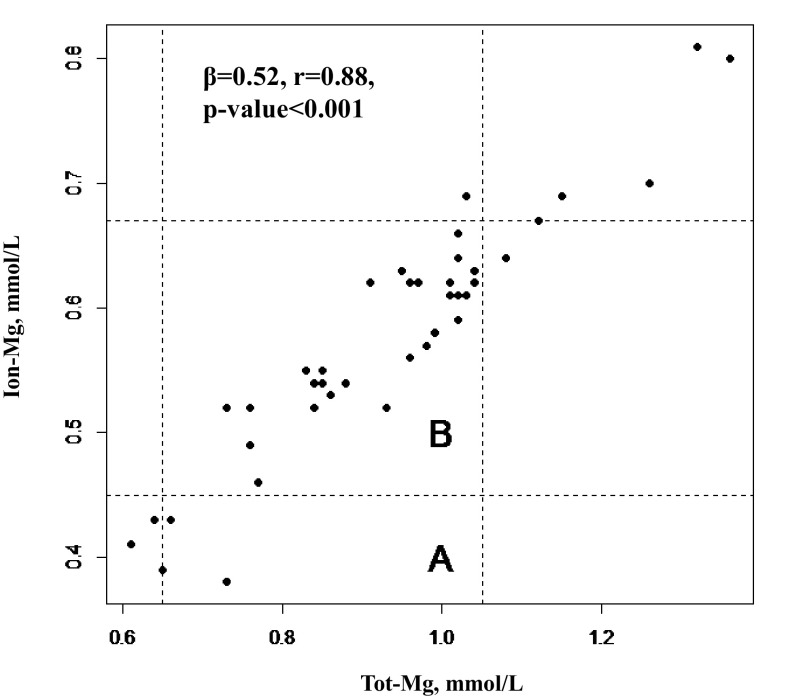
Relationship between serum tot-Mg and ion-Mg. Reference ranges of total (0.65–1.05 mmol/L) and ionized magnesium (0.45–0.67 mmol/L) are shown and delimited with dashed lines. **a** Normal range for ion-Mg, **b** normal range for tot-Mg. On the figure are also reported the results of the linear regression

The association between serum tot-Mg and ion-Mg is depicted in a scatter plot in Fig. 3. In the same figure the result of the linear regression, showing a significant association between serum tot-Mg and ion-Mg (β coefficient 0.52; *r* 0.88; *p* < 0.001) can also be seen. Dashed lines, delimiting the reference range values for ion-Mg and serum tot-Mg, confirm that 10% of patients considered in range with the serum tot-Mg, are not in range with the ionized one and that a subgroup of patients with tot-Mg values above the upper range have false hyper-magnesemie.

We then performed a stepwise multiple regression with backward elimination to determine the effect of the potential explanatory variables on ion-Mg considering first the subset (26 subjects) of normo-albuminemic patients (albumin > 34 g/L) and thereafter the subset of hypo-albuminemic patients (16 subjects) (albumin < 35 g/L). The following covariates were significantly associated with ion-Mg: K^+^ (*p* < 0.001), Ca^2+^ (*p* = 0.004), HCO^3−^ (*p* = 0.002), serum tot-Mg (*p* < 0.001) and Hb (*p* = 0.021) (Table 2). The final regression equation, algebraically simplified in order to calculate ion-Mg, for normo-albuminemic patients was: ion-Mg = tot-Mg/2-K^+^/50 + ion-Ca^2+^-HCO^3−^/100 (Table 2). In the subset of hypo-albuminemic patients ion-Mg resulted significantly associated with albumin (*p* < 0.001) and tot-Mg (*p* = 0.021). The final regression equation was ion-Mg = tot-Mg/2 + albumin/100 (Table 2).

**Table 2:** Stepwise multiple linear regression to determine the effect of selected variables on ion-Mg

Variables	β-coefficient	Standard error	*p* value
Normo-albuminemic patients (no. 26)			
K^+^	− 0.21	0.005	0.001
Ion-Ca^2+^	0.197	0.05	0.004
HCO^3−^	− 0.006	0.001	0.002
Tot-Mg	0.448	0.363	≤0.001
Hemoglobin	0.0008	0.0003	0.021
Hypo-albuminemic Patients (no. 16)			
Tot-Mg	0.47	0.046	0.021
Albumin	0.007	0.002	≤0.001

The final regression equations, algebraically simplified, for normo- and for hypo-albuminemic patients, are respectively: ion-Mg = tot-Mg/2-K/50 + calcium/5-HCO^3−^/100, ion-Mg = tot-Mg/2 + albumin/100

Considering the known association between serum albumin and diabetes [19], we investigated in a post hoc analysis the albumin levels comparing diabetic and non-diabetic patients and no differences were found: 33.6 g/L (31.7–35.5) vs. 34.3 g/L (31.6–36.9) *p* = 0.640. In the multiple linear regression analysis also, diabetes was not associated with albumin (β coefficient 0.63; −2.91 to 4.18; *p* = 0.720).

**Fig. 4:**
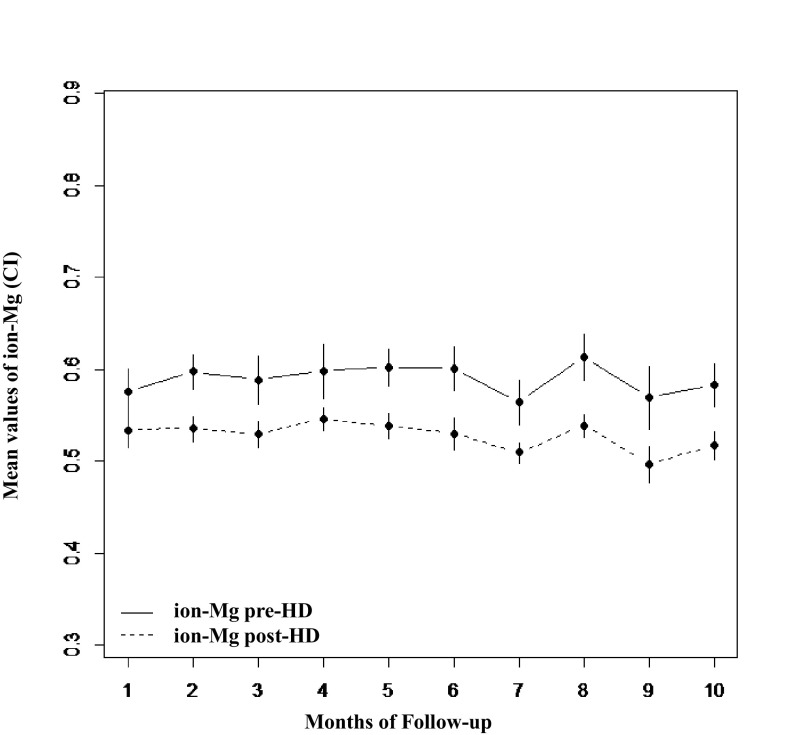
Ion-Mg variability in the longitudinal phase. Variability across months of observation of pre- vs. post-HD ion-Mg in the longitudinal phase. Error bars express the 95% confidence interval

In the longitudinal phase the amount of patients in normo-, hypo- and hyper-magnesemic based on the values of ion-Mg; were respectively: 88, 2 and 10% in pre-HD; and 95, 5 and 0% in post-HD. The variability of pre- and post-HD ion-Mg during the months of follow-up is depicted in Fig. 4.

**Fig. 5:**
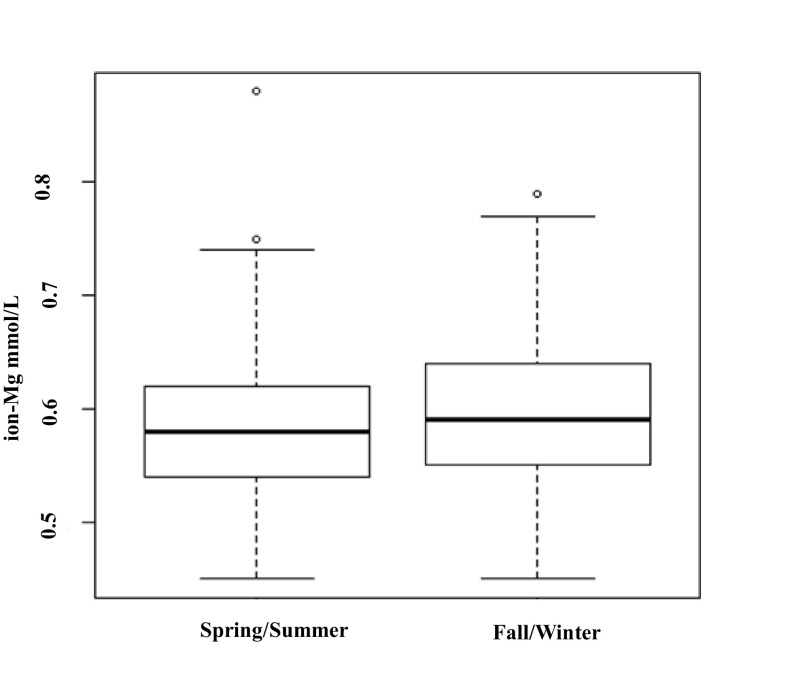
Seasonal variability of ion-Mg

We then investigated the seasonal variability of ion-Mg using a linear regression model in which we included binary indicators for the season (summer vs. winter) and the patient’s indicator as a fixed effect. The *p* value for the null effect of season on the likelihood ratio test was 0.027 (Fig. 5).

Inter- and intra-individual variabilities of ion-Mg was evaluated based on coefficient of variation (CV) as depicted in Table 3. In the longitudinal phase, we analyzed the number and the rate of hypo-magnesemic events across the months under analysis to identify a subset of patients with higher and lower magnesium level variability. The two groups (higher vs. lower variability) significantly differed for: gender (females: 64 vs. 34%; *p* < 0.001); age (66 vs. 77 years; *p* < 0.001); Urea pre-HD (26.3 vs. 21.2 mmol/L; *p* < 0.001); nPCR 0.92 vs 0.77 g/kg day; *p* < 0.001) and PTH (42.3 vs 28.5 pmol/L; *p* < 0.001) (Table 4).

**Table 3:** Intra- and inter-individual variabilities of ion-Mg

Subjects	Mean of 10/HD/sessions	CV	Mean of 10/HD/sessions	CV	Mean of 10/HD/sessions	CV
Mg ion pre-HD Mg ion post HD delta Mg						
Paz 1	0.522	0.054	0.536	0.054	−0.014	2.26
Paz 2	0.625	0.040	0.566	0.040	0.059	0.455
Paz 3	0.574	0.036	0.466	0.036	0.108	0.257
Paz 4	0.650	0.036	0.562	0.036	0.087	0.524
Paz 5	0.560	0.041	0.552	0.041	0.044	0.437
Paz 6	0.572	0.061	0.502	0.061	0.067	0.988
Paz 7	0.569	0.088	0.514	0.088	0.060	0.707
Paz 8	0.555	0.022	0.511	0.022	0.042	0.869
Paz 9	0.703	0.042	0.520	0.042	0.095	0.536
Paz 10	0.664	0.028	0.569	0.028	0.183	0.287
Paz 11	0.580	0.027	0.531	0.027	0.095	0.550
Paz 12	0.676	0.060	0.547	0.060	0.128	0.404
Paz 13	0.573	0.050	0.551	0.050	0.011	3.538
Paz 14	0.603	0.038	0.559	0.038	0.041	0.760
Paz 15	0.543	0.096	0.481	0.096	0.059	0.390
Paz 16	0.588	0.046	0.543	0.046	0.044	0.422
Paz 17	0.529	0.043	0.500	0.043	0.027	0.947
Paz 18	0.618	0.046	0.547	0.046	0.064	0.491
Paz 19	0.487	0.147	0.444	0.147	0.046	1.189
Paz 20	0.506	0.038	0.466	0.038	0.040	0.645
Paz 21	0.623	0.056	0.569	0.056	0.054	0.656
Paz 22	0.616	0.035	0.533	0.035	0.080	0.331
Paz 23	0.543	0.062	0.540	0.062	0.003	10.31
Paz 24	0.574	0.056	0.542	0.056	0.036	0.658
Paz 25	0.590	0.033	0.491	0.033	0.098	0.333
Paz 26	0.549	0.044	0.520	0.044	0.032	1.134
Paz 27	0.690	0.075	0.551	0.075	0.139	0.690
Inter-individual CV		0.116		0.082		0.916

**Table 4:** Differences between patients based on variability (group with high variability vs group with low variability)

Variables	Higher variability	Lower variability	*p* value
Females (%)	64.3%	38.4%	<0.001
Age (years)	66 ± 8	77 ± 8	<0.001
Urea pre-HD (mmol/L)	26.3 ± 0.5	21.2 ± 0.4	<0.001
Urea post-HD (mmol/L)	5.5 ± 1.8	5.9 ± 1.5	0.028
nPCR (g/Kg)	0.92 ± 0.1	0.77 ± 0.1	<0.001
Phosphates (mmol/L)	1.8 ± 0.04	1.5 ± 0.03	<0.001
K^+^ (mmol/L)	5.1 ± 0.5	5.0 ± 0.6	NS
Weight (kg)	71.3 ± 1.0	69.9 ± 1.2	NS
Hb (g/L)	22.02 ± 2.8	22.01 ± 0.01	NS
Ferritin (mg/dL)	426.6 ± 51.1	633.5 ± 58.5	NS
PTH (pmol/L)	46.3 ± 4	28.5 ± 3	<0.001
25-OH-vitamin D (ng/mL)	42.3 ± 3.5	46.3 ± 2.4	NS

## Discussion

In this study, we investigated the add-on value of assessing serum ionized Mg compared to total Mg in detecting HD patients with pathological magnesium levels and in evaluating, without confounding factors, the inter- and intra-individual variabilities. The ultimate goal of the investigation was to identify patients potentially at risk of hypermagnesemia, if exposed to higher dialysate magnesium concentrations.

Measurement of ion-Mg has been found to be useful in several clinical circumstances [17] and various techniques, not routinely in use, have been developed for its assessment [16]. Previous studies have also investigated the sensitivity of serum ion-Mg and tot-Mg assays in detecting magnesium overload but with conflicting results [20–22].

Our study suggests that 10% of the patients, otherwise considered as normo-magnesemic, can be reclassified as hyper- and hypo-magnesemic on the basis of ion-Mg and that a small subgroup of patients with tot-Mg levels above the upper normal range are false hyper-magnesemic. Confirming the results of previous studies, the ion-Mg fraction in HD patients represents 60–70% of total magnesium [23].

Furthermore two simple formulas, for normo- and hypo-albuminemic patients, aimed at calculating ion-Mg on the basis of tot-Mg, were extrapolated using the study data. The use of our formulas elaborated in the hemodialysis setting could enable more appropriate decision making in this highly peculiar population.

The findings of previous studies indicate that ion-Mg is affected by significant variability related mainly to the circadian rhythm [24]. This physiological variability associated with the numerous confounding factors specific to CKD and hemodialysis are further arguments for measuring ion-Mg in cases of using dialysates with magnesium levels at the upper limit of the normal distribution. Intra-individual variation in serum tot-Mg ranges between 3.4 and 4.7% [22, 25]. In our study a high intra- and inter-individual variabilities in ion-Mg was also found. As stated before, for several electrolytes cyclical rhythms, circadian, monthly, or seasonal, were previously observed. In our study a seasonal variability of ion-Mg was recorded, with higher levels of ion-Mg during the winter. Similar results, outside the CKD setting, were previously obtained using an experimental animal model [26]. Knowing that the winter diet is expected to contain less magnesium and that a lower exposure to calcidiol should occur in the same months, other factors like increased sweating in summer could be related to the seasonal differences found.

Knowledge of the inter- and intra-individual variabilities over time, is essential in our opinion, when the decision of increasing magnesium content in the dialysate is made. Patients with high variability could in fact represent a population potentially at risk of hyper-magnesemia. In our study, the subset of patients with high variability are younger females with a better nutritional status (higher levels of nPCR and urea); a condition that has been associated with higher total body magnesium content [27, 28]. Our study, however, has two main limitations; the small sample size and its observational nature. Therefore, an interventional study with different magnesium dialysate concentrations has been planned.

## Conclusions

Determination of serum ion-Mg could be useful in unmasking unrecognized hyper- and hypo-magnesemic and false hyper-magnesemic patients. Ion-Mg is characterized by high intra- and inter-individual variabilities, particularly in younger women and those with better nutrition.

In the hemodialysis population, when planning a systematic increase in the dialysate magnesium concentration aimed to counteract both, hemodynamic instability during the session and vascular calcifications, efficiently identifying subjects at risk of hypermagnesemia could help to avoid unwanted side effects and risks.

## Abbreviations

Ion-Mg: Ionized magnesium
Tot-Mg: Total serum magnesium
HD: Hemodialysis
CKD: Chronic kidney disease
HCO^3−^: Serum bicarbonate
Ca^2+^: Ionized calcium
K^+^: Serum potassium
Na^+^: Serum sodium
P: Phosphate
Hb: Hemoglobin
PTH: Parathormone
